# Retraction Note: Simulation of hybridized nanofluids flowing and heat transfer enhancement via 3-D vertical heated plate using finite element technique

**DOI:** 10.1038/s41598-024-59124-1

**Published:** 2024-04-15

**Authors:** Muhammad Bilal Hafeez, Marek Krawczuk, Hasan Shahzad, Amjad Ali Pasha, Mohammad Adil

**Affiliations:** 1 Faculty of Mechanical Engineering and Ship Technology, Institute of Mechanics and Machine Design, Gdansk University of Technology, Narutowicza 11/12, 80-233 Gdańsk, Poland; 2 Faculty of Materials and Manufacturing, College of Mechanical Engineering and Applied Electronics Technology, Beijing University of Technology, Beijing, China; 3 Aerospace Engineering Department, King Abdulaziz University, 21589 Jeddah, Saudi Arabia; 4 Mechanical Engineering Program, Physical Science and Engineering Division, King Abdullah University of Science and Technology, 23955-6900 Thuwal, Saudi Arabia; 5 KAUST Clean Combustion Research Center, King Abdullah University of Science and Technology, 23955-6900 Thuwal, Saudi Arabia

Retraction of: *Scientific Reports* 10.1038/s41598-022-15560-5, published online 08 July 2022

The Editors have retracted this Article.

After publication of this Article concerns were raised over relevance of some of the references to the content they were cited to support. Editors requested that, in addition to clarification about these concerns, the Authors provide the underlying code for the simulations. An expert post-publication peer review of the provided code showed that it is not suited to reproduce the reported results. Therefore, the Editors lost confidence in the results and conclusions of the Article.

Marek Krawczuk and Amjad Ali Pasha do not agree with the retraction. Muhammad Bilal Hafeez did not explicitly state whether or not they agreed with this retraction. Hasan Shahzad and Mohammad Adil did not respond to the correspondence from the Editors about the retraction.

